# KYP-2047, an Inhibitor of Prolyl-Oligopeptidase, Reduces GlioBlastoma Proliferation through Angiogenesis and Apoptosis Modulation

**DOI:** 10.3390/cancers13143444

**Published:** 2021-07-09

**Authors:** Sarah Adriana Scuderi, Giovanna Casili, Alessio Ardizzone, Stefano Forte, Lorenzo Colarossi, Serena Sava, Irene Paterniti, Emanuela Esposito, Salvatore Cuzzocrea, Michela Campolo

**Affiliations:** 1 Department of Chemical, Biological, Pharmaceutical and Environmental Sciences, University of Messina, Viale Ferdinando Stagno D’ Alcontres, 31-98166 Messina, ME, Italy; sarahadriana.scuderi@unime.it (S.A.S.); gcasili@unime.it (G.C.); aleardizzone@unime.it (A.A.); ipaterniti@unime.it (I.P.); salvator@unime.it (S.C.); michela.campolo@unime.it (M.C.); 2 IOM Ricerca Srl, Via Penninazzo 11, 95029 Viagrande, CT, Italy; stefano.forte@grupposamed.com; 3 Istituto Oncologico del Mediterraneo, Via Penninazzo 7, 95029 Viagrande, CT, Italy; lorenzo.colarossi@grupposamed.com (L.C.); serena.sava@grupposamed.com (S.S.)

**Keywords:** glioblastoma (GB), prolyl-oligopeptidase (POP), vascular endothelial growth factor (VEGF), transforming growth factor-β (TGF-β), angiopoietin (Ang), endothelial nitric oxide synthase (eNOS)

## Abstract

**Simple Summary:**

Glioblastoma (GB) is the most aggressive brain tumor characterized by necrosis, excessive proliferation, and invasiveness. Despite relevant progress in conventional treatments, the survival rate for patients with GB remains low. The present study investigated the potential effect of KYP-2047, an inhibitor of the prolyl-oligopeptidase (POP or PREP), in an in vivo U87-xenograft model and in an in vitro study on human GB cells. This study demonstrated the abilities of KYP-2047 to counteract and reduce GB progression through angiogenesis and apoptosis modulation.

**Abstract:**

Glioblastoma (GB) is the most aggressive tumor of the central nervous system (CNS), characterized by excessive proliferation, necrosis and invasiveness. The survival rate for patients with GB still remains low. Angiogenesis and apoptosis play a key role in the development of GB. Thus, the modulation of angiogenesis and apoptosis processes represent a possible strategy to counteract GB progression. This study aimed to investigate the potential effect of KYP-2047, an inhibitor of the prolyl-oligopeptidase (POP), known to modulate angiogenesis, in an in vivo U87-xenograft model and in an in vitro study on human GB cells. Our results showed that KYP-2047 at doses of 2.5 mg/kg and 5 mg/kg was able to reduce tumor burden in the xenograft-model. Moreover, KYP-2047 significantly reduced vascular endothelial-growth-factor (VEGF), angiopoietins (Ang) and endothelial-nitric-oxide synthase (eNOS) expression. In vitro study revealed that KYP-2047 at different concentrations reduced GB cells’ viability. Additionally, KYP-2047 at the concentrations of 50 µM and 100 µM was able to increase the pro-apoptotic protein Bax, p53 and caspase-3 expression whereas Bcl-2 expression was reduced. Thus, KYP-2047 could represent a potential therapeutic treatment to counteract or reduce GB progression, thanks its abilities to modulate angiogenesis and apoptosis pathways.

## 1. Introduction

Gliomas are the main neoplastic diseases affecting the central nervous system (CNS) [1]. Among gliomas, glioblastoma (GB) is the most common primary malignant tumor of CNS, with an incidence of about 3–4 cases per 100,000 people per year [2]. GB is classified by the World Health Organization (WHO) as grade IV astrocytoma, characterized by poorly differentiated neoplastic astrocytes with high mitotic activity, necrosis and vascular proliferation [2]. GB occurs more frequently in mature people aged between 45 and 75 years with a higher incidence in men than in women, associated with a poor quality of life [3]. GB is characterized by abnormal angiogenesis, apoptosis alteration and invasiveness [4]. Genome-wide expression studies in glioblastomas revealed that GB is associated with chromosomic alterations which can include deletions, amplifications or mutations which contribute to the development of GB [5]. In addition to genetic risk factors, other risk factors involved in the development of GB have been identified, such as exposure to ionizing radiation, ultraviolet rays, smoke, and pesticides [3,6]. The symptomatology of GB is varied, as it is related to the location and degree of infiltration of the tumor mass. Currently, standard treatment for GB includes surgical removal of the tumor, followed by the concomitant administration of chemotherapeutic agents such as temozolomide (TMZ) and radiotherapy [3]. However, the survival rate for patients with GB still remains low [7]; consequently, the identification of new therapeutic targets and new molecules able to reduce or arrest the progression of GB represents an important goal for cancer research. Many studies have focused on the role of angiogenesis and apoptosis in the development of GB [8,9]. It has been proposed that therapeutic resistance of GB is due to an up-regulation of anti-apoptotic proteins such as Bcl2 and a downregulation of pro-apoptotic proteins, leading to activation of oncogenes that promote tumor cell survival [9]. Moreover, also angiogenesis represents a key event for tumor growth and progression [10]; in fact, it has been demonstrated that several angiogenic factors such as vascular endothelial growth factor (VEGF) and angiopoietins (Ang) are up-regulated in GB that generate highly permeable and functionally immature blood vessels which contribute to tumor growth [8,10]. Recently, different studies have focused on the effect of KYP-2047 [11,12], a specific and potent inhibitor of the prolyl-oligopeptidase (POP or PREP), a serine protease involved in the angiogenesis process [11,12]. POP is present both in the brain and in peripheral tissues; it is involved in the hydrolysis of proline and in many other physiological functions [13]. KYP-2047 demonstrated the ability to modulate the angiogenesis process, but also cell cycle and differentiation [11,12,13]. Therefore, considering the key roles of angiogenesis and apoptosis in GB pathology, the aim of this study was to investigate the potential effect of KYP-2047 in an in vivo U87-xenograft model and in vitro model on human GB cells to counteract or reduce GB progression. 

## 2. Materials and Methods

### 2.1. In Vivo Studies

#### 2.1.1. Cell Line

The human GB cell line U-87 (U-87MG ATCC^®^ HTB-14™ Homo sapiens brain Likely glioblastomas) was obtained from ATCC (American Type Culture Collection, Rockville, MD, USA). U-87 cells were cultured in 75 cm^2^ flask with respectively Dulbecco’s modified Eagle’s medium (DMEM—Sigma-Aldrich^®^ Catalog No. D5030; St. Louis, MO, USA) supplemented with antibiotics (penicillin 1000 units—streptomycin 0.1 mg/L, Sigma-Aldrich^®^ Catalog No. P4333; St. Louis, MO, USA), L-glutamine (GlutaMAX™, ThermoFisher Scientific^®^ Catalog No. 35050061; Waltham, MA, USA) and 10% (*v*/*v*) fetal bovine serum (FBS, Sigma-Aldrich^®^ Catalog No. 12103C St. Louis, MO, USA) in a humidified atmosphere containing 5% CO_2_ at 37 °C.

#### 2.1.2. Animals

Wild-type nude male mice C57BL/6J were purchased from Jackson Laboratory (Bar Harbor, Hancock, ME, USA) and housed in microisolator cages under pathogen-free conditions on a 12 h light/12 h dark schedule for a week. Animals were fed a standard diet and water ad libitum. Animal experiments were in compliance with Italian regulations on protection of animals used for experimental and other scientific purposes (DM 116192) as well as European Union (EU) regulations (OJ of EC L 358/1 18 December 1986). 

#### 2.1.3. Experimental Design

The Xenograft tumor model was performed as previously described by Deng et al. [14]. The mice were inoculated subcutaneously with 3 × 10^6^ human glioblastoma U-87 cells in 0.2 mL of phosphate buffered saline (PBS) and 0.1 mL matrigel (BD Bioscience, Bedford, MA, USA). Animals were treated with KYP-2047 at doses of 1 mg/kg, 2.5 mg/kg and 5 mg/kg every three days from day 7. KYP-2047 was dissolved in PBS with 0.001% of dimethyl sulfoxide (DMSO). After tumor cell inoculation, animals were monitored daily for morbidity and mortality [15]. At the thirty-fifth day, the animals were sacrificed and their tumors were excised and processed for analysis. Tumor volumes were measured non-invasively by using an electronic calliper. The tumor burden was calculated using the following formula: 0.5 × length × width. The tumor size was measured every four days for 28 days. The tumor volume was calculated using an empirical formula, V = 1/2 × ((the shortest diameter) 2 × (the longest diameter)). The experiments were performed three times to verify the data, using 25 animals for each experimental group. 

Experimental groups:

The mice were randomly divided into four groups, as described below:

1. Control group (vehicle): weekly intravenous (IV) administration of saline.
2. Control group + KYP-2047 1 mg/kg: intraperitoneal (ip) administration of KYP-2047 1 mg/kg dissolved in PBS every three days from day 7.
3. Control group + KYP-2047 2.5 mg/kg: intraperitoneal (ip) administration of KYP-2047 2.5 mg/kg dissolved in PBS every three days from day 7.
4. Control group + KYP-2047 5 mg/kg: intraperitoneal (ip) administration of KYP-2047 5 mg/kg dissolved in PBS every three days from day 7.

Furthermore, the control group + KYP-2047 1 mg/kg was only subjected to histological evaluation, mean tumor burden and mean tumor weight, because it did not induce any beneficial effect; therefore, we decided to continue analyzing only KYP-2047 2.5 mg/kg and 5 mg/kg groups.

#### 2.1.4. Histological Evaluation

Histological evaluation was performed as previously described by Esposito et al. [16]. Tumor samples were fixed with 10% neutral formalin, embedded in paraffin, and sectioned at 7 µm. Sections were deparaffinized with xylene and stained with hematoxylin and eosin. The slides were analyzed by a pathologist blinded to the treatment groups. All sections were analyzed using an Axiovision microscope (Zeiss, Milan, Italy).

#### 2.1.5. Western Blot Analysis

Tumor samples from each mouse were suspended in extraction Buffer A (0.2 mM PMSF, 0.15 mM pepstatin A, 20 mM leupeptin, 1 mM sodium orthovanadate), homogenized at the highest setting for 2 min, and centrifuged at 12,000× *g* rpm for 4 min at 4 °C. Supernatants are the cytosolic fraction, whereas the pellets, containing enriched nuclei, were resuspended in Buffer B (1% Triton X-100, 150 mM NaCl, 10 mM TrisHCl pH 7.4, 1 mM EGTA, 1 mM EDTA, 0.2 mM PMSF, 20 mm leupeptin, 0.2 mM sodium orthovanadate) and centrifuged at 12,000× *g* rpm for 10 min at 4 °C; supernatants are the nuclear fraction. Protein concentration was estimated by the Bio-Rad protein assay using bovine serum albumin as standard. Then, tumor samples, in equal amounts of protein, were separated on 12% SDS-PAGE gel and transferred to nitrocellulose membrane as previously described [17]. The following primary antibodies were used: anti-vascular endothelial growth factor (VEGF) (1:500; Santa Cruz Biotechnology, Dallas, TX, USA; sc-7269); anti-endothelial nitric oxide synthase (eNOS) (1:500; Santa Cruz Biotechnology, Dallas, TX, USA; sc-376751); anti-angiopoietin 1 (Ang1) (1:500; Santa Cruz Biotechnology, Dallas, TX, USA; sc-517593); anti-angiopoietin 2 (Ang2) (1:500; Santa Cruz Biotechnology, Dallas, TX, USA; sc-74403); anti-Ki-67 (1:500; Santa Cruz Biotechnology, Dallas, TX, USA; sc-23900); anti-Bax (1:500; Santa Cruz Biotechnology, Dallas, TX, USA; sc-7480); anti-Bcl2 (1:500; Santa Cruz Biotechnology, Dallas, TX, USA; sc-7382). Antibody dilutions were made in PBS/5% *w*/*v* nonfat dried milk/0.1% Tween-20 (PMT) and membranes incubated overnight at 4 °C. Membranes were then incubated with secondary antibody (1:2000, Jackson ImmunoResearch, West Grove, PA, USA) for 1 h at room temperature. To ascertain that those blots were loaded with equal amounts of protein lysate, they were also incubated with β-actin antibody (for cytosolic fraction 1:500; Santa Cruz Biotechnology, Dallas, TX, USA; sc-8432) or lamin A/C (for nuclear fraction 1:500, Santa Cruz Biotechnology, Dallas, TX, USA; sc-376248). Signals were detected with an enhanced chemiluminescence (ECL) detection system reagent according to the manufacturer’s instructions (Thermo Fisher, Waltham, MA, USA). The relative expression of the protein bands was quantified by densitometry with BIORAD ChemiDocTMXRS + software. 

#### 2.1.6. Immunohistochemical Localization of Vascular Endothelial-Growth-Factor (VEGF), Endothelial Nitric Oxide Synthase (eNOS), CD34, Ki-67, Bcl2 and Caspase-3

Immunohistochemical localization was performed as previously described by Esposito et al. [16]. Slides were incubated overnight using the following primary antibodies: VEGF (Santa Cruz Biotechnology, Dallas, TX, USA; 1:100 in PBS, *v*/*v*; sc-7269), eNOS (Santa Cruz Biotechnology, Dallas, TX, USA, 1:100 in PBS, *v*/*v*; sc-376751) anti-Bcl2 (1:100; Santa Cruz Biotechnology, Dallas, TX, USA; sc-7382); anti-caspase-3 (1:100, Santa Cruz Biotechnology, Dallas, TX, USA; sc-56053); anti-Ki-67 (1:100; Santa Cruz Biotechnology, Dallas, TX, USA; sc-23900); anti-CD34 (1:100; Santa Cruz Biotechnology, Dallas, TX, USA; sc-74499). At the end of the incubation with the primary antibodies, the sections were abundantly washed with PBS and incubated with a secondary antibody (Santa Cruz Biotechnology, Dallas, TX, USA) for 1 h at room temperature. The reaction was revealed by a chromogenic substrate (brown DAB), and counterstaining with NUCLEAR FAST-RED. The percentage of positive staining was measured using a computerized image analysis system (Leica QWin V3, Cambridge, UK). The images were acquired using an optical microscope (Zeiss, Axio Vision, Feldbach, Schweiz). For immunohistochemistry, the images were shown at a magnification of 20 × (50 μm of the bar scale).

#### 2.1.7. Caspase-3 Activity Measurement

Caspase-3 activity in tumor lysate was measured using a colorimetric Assay Kit (cat#ab39401, Abcam, Cambridge, UK) as suggested by manufacturer’s instruction.

#### 2.1.8. RNA Isolation and Quantitative Real-Time Polymerase Chain Reaction (RT-qPCR)

Total RNA of tumor samples was isolated using TRIzol reagent (Invitrogen, Carlsbad, CA, USA) according to the manufacturer’s instructions. RNA isolation was performed as previously described by Weinert et al. [18]. First-strand cDNA obtained from RNA samples was stored at −80 °C until use.

The mRNA expression levels of VEGF and eNOS in each sample, was measured using Power Up Sybr Master Mix (Applied Biosystems) and a QuantStudio Flex Real-Time Polymerase Chain Reaction (PCR) System (Applied Biosystems) [19]. The primer used for reverse transcriptase PCR were for VEGF: forward 5′-GAGCAGAAGTCCCATGAAGTGA-3′ and reverse 5′-CACAGGACGGCTTGAAGATGT-3′; eNOS: forward 5′-CCTGTGAGACCTT CTGTGTGG-3′ and reverse 5′-GGATCAGACCTGGCAGCAACT-3′. The mRNA expression levels were normalized to that of glyceraldehyde-3-phosphate dehydrogenase (GAPDH): forward: 5′-GGGCTGGCATTGCTCTCA-3′, reverse: 5′-TGCTGTAGCGTATTCATTG-3′. Each sample was analyzed in triplicate, and all tests were repeated at least three times. 

#### 2.1.9. Enzyme-Linked Immunosorbent Assay (ELISA) Kit 

An enzyme-linked immunosorbent assay (ELISA) kit was performed to evaluate PREP expression in serum of each mice using Mouse PREP ELISA kit (cat#Q9QUR6 RayBiotech, Peachtree Corners, GA, USA) as suggested by manufacturer’s instructions. The serum of each animal was collected and measured by ELISA kit once a week.

#### 2.1.10. Immunofluorescence Assay 

Immunofluorescence staining was performed as previously described by Campolo et al. [20]. Tumor samples were collected and processed for immunofluorescence staining. Tissue sections of 7 μm were incubated with the following primary antibody anti-CD34 at 37 °C overnight (1:100; Santa Cruz Biotechnology, Dallas, TX, USA; sc-74499). Then, tissue sections were washed with PBS and incubated with secondary antibody anti-mouse Alexa Fluor-488 antibody (1:1000 *v*/*v*, Molecular Probes, Altrincham, UK) for 1 h at 37 °C. For nuclear staining, 4′,6′-diamidino-2-phenylindole (DAPI; Hoechst, Frankfurt, Germany) (2 μg/mL) in PBS was added. Sections were observed and photographed at 40× magnification using a Leica DM2000 microscope.

### 2.2. In Vitro Studies

#### 2.2.1. Cell Lines

U-87 MG (U-87 MG ATCC^®^ HTB-14™ Homo sapiens brain likely glioblastomas), U-138MG (U-138 MG ATCC^®^ HTB-16™ Homo sapiens brain glioblastoma IV grade), A-172 (A-172 ATCC^®^ CRL-1620™ Homo sapiens brain glioblastoma) were obtained from ATCC (American Type Culture Collection, Rockville, MD, USA). The human GB cell lines were seeded in 75 cm^2^ flask with respectively Dulbecco’s modified Eagle’s medium (DMEM—Sigma-Aldrich^®^ Catalog No. D5030; St. Louis, MO, USA) supplemented with antibiotics (penicillin 1000 units—streptomycin 0.1 mg/L, Sigma-Aldrich^®^ Catalog No. P4333; St. Louis, MO, USA), L-glutamine (GlutaMAX™, ThermoFisher Scientific^®^ Catalog No. 35050061; Waltham, MA, USA) and 10% (*v*/*v*) FBS (Sigma-Aldrich^®^ Catalog No. 12103C St. Louis, MO, USA) in a humidified atmosphere containing 5% CO_2_ at 37 °C.

#### 2.2.2. Cell Treatment 

Human GB cells were plated on 96-well plates at a density of 4 × 10^4^ cells/well to a final volume of 150 μL. After 24 h, GB cells were treated with KYP-2047 (Sigma-Aldrich^®^) for 24 h at increasing concentrations 0.01 μM, 0.1 μM, 0.5 μM, 1 μM, 10 μM, 30 μM, 50 μM and 100 μM dissolved in PBS.

Experimental Groups:

1. Control group (Ctr): human GB cell lines U-87, U-138 and A-172;
2. KYP-2047 0.01 μM group: GB cells treated with KYP-2047 0.01 μM;
3. KYP-2047 0.1 μM group: GB cells treated with KYP-2047 0.1 μM;
4. KYP-2047 0.5 μM group: GB cells treated with KYP-2047 0.5 μM;
5. KYP-2047 1 μM group: GB cells treated with KYP-2047 1 μM;
6. KYP-2047 10 μM group: GB cells treated with KYP-2047 10 μM;
7. KYP-2047 30 μM group: GB cells treated with KYP-2047 30 μM;
8. KYP-2047 50 μM group: GB cells treated with KYP-2047 50 μM;
9. KYP-2047 100 μM group: GB cells treated with KYP-2047 100 μM;

The experiments were repeated three times to verify the data.

For western blot analysis and immunofluorescence assay on U-87, A-172 and U-138 cells, we decided to continue to analyze only KYP-2047 at the concentrations of 50 μM and 100 μM because represented the most cytotoxic concentrations revealed by MTT assay. 

#### 2.2.3. Cell Viability Assay

Cell viability assay on U-87, U-138 and A-172 cells were performed using a mitochondria-dependent dye for live cells (tetrazolium dye; MTT) to formazan [20]. GB cells were pre-treated with increasing concentrations of KYP-2047 for 24 h. After 24 h, cells were incubated at 37 °C with MTT (0.2 mg/mL) for 1 h. The medium was removed, and the cells lysed with dimethyl sulfoxide (DMSO) (100 μL). The extent of reduction in MTT to formazan was quantified by measurement of optical density (OD) at 550 nm with a microplate rider.

#### 2.2.4. Western Blot Analysis

Western blot analysis on U-87, A-172 and U-138 cell lysates was performed as previously described by Campolo et al. [20]. Human GB cells were washed with ice-cold PBS harvested and resuspended in Tris-HCl 20 mM pH 7.5, NaF 10 mM, 150 μL NaCl, 1% Nonidet P-40 and protease inhibitor cocktail (Roche). After 40 min, cell lysates were centrifuged at 16,000× *g* for 15 min at 4 °C. Protein concentration was estimated by the Bio-Rad protein assay using bovine serum albumin as standard. Samples were then heated at 95 °C for 5 min and equal amounts of protein separated on a 10–15% SDS-PAGE gel and transferred to a PVDF membrane (Immobilon-P). The membranes were incubated overnight at 4 °C with primary antibodies: anti-Bax (1:500; Santa Cruz Biotechnology, Dallas, TX, USA; sc-7480); anti-Bcl2 (1:500; Santa Cruz Biotechnology, Dallas, TX, USA; sc-7382); anti-p53 (1:500; Santa Cruz Biotechnology, Dallas, TX, USA; sc-126). To ascertain that blots were loaded with equal amounts of protein lysate, they were also incubated with the antibody β-actin for cytosolic fraction (1:500; Santa Cruz Biotechnology; Dallas, TX, USA. sc-8432) and lamin A/C for nuclear fraction (1:500; Santa Cruz Biotechnology; Dallas, TX, USA, sc-376248). Signals were detected with enhanced chemiluminescence (ECL) detection system reagent according to the manufacturer’s instructions (Thermo Fisher, Waltham, MA, USA). The relative expression of the protein bands was quantified by densitometry with BIORAD ChemiDocTMXRS + software. 

#### 2.2.5. Immunofluorescence Assay for Transforming Growth Factor-β (TGF-β) and Caspase-3

Immunofluorescence assay was performed on U-87, A-172 and U-138 cells as previously described by Donaldson [21]. GB cells on glass cover slips were rinsed briefly in phosphate-buffered saline (PBS:0.15 M NaCl, 10 mM Na_2_HPO_4_, pH 7.4), permeabilized in 0.2% Triton X-100/PBS and blocked with 10% goat serum. The cells were stained overnight (O/N) at 4 °C with primary antibodies: anti-transforming growth factor-β (TGFβ, 1:50, Santa Cruz Biotechnology, Dallas, TX, USA; sc-130348) and anti-caspase-3 (1:50, Santa Cruz Biotechnology, Dallas, TX, USA; sc-56053). At the end of the incubation with the primary antibody, the sections were abundantly washed with PBS and incubated with a secondary antibody anti-mouse Alexa Fluor-488 antibody (1:1000 *v*/*v* Molecular Probes, UK) for 1 h at 37 °C. Sections were washed in PBS and for nuclear staining 4′,6′-diamidino-2-phenylindole (DAPI; Hoechst, Frankfurt; Germany) 2 μg/mL in PBS was added. Sections were observed and photographed at 40× magnification using a Leica DM2000 microscope (Leica, Axio Vision, Feldbach, Schweiz). All images were digitalized at a resolution of 8 bits into an array of 2560 × 1920 pixels. Optical sections of fluorescence specimens were obtained using a HeNe laser (543 nm), an ultraviolet (UV) laser (361–365 nm), and an argon laser (458 nm) at a 1 min, 2 s scanning speed with up to eight averages; 1.5 μm sections were obtained using a pinhole of 250. Contrast and brightness were established by examining the most brightly labeled pixels and applying settings that allowed clear visualization of structural details while keeping the highest pixel intensities close to 250.

### 2.3. Materials

KYP-2047 and all other chemicals were obtained by Sigma-Aldrich (Milan, Italy). All stock solutions were prepared in non-pyrogenic saline (0.9% NaCl, Baxter, Milan, Italy).

### 2.4. Statistical Analysis 

All values are expressed as mean ± standard error of the mean (SEM) of “n” observations. Each analysis was performed three times with three samples replicates for each one. The results were analyzed by one-way analysis of variance (ANOVA) followed by a Bonferroni post hoc test for multiple comparisons. A *p*-value of less than 0.05 was considered significant.

## 3. Results

### 3.1. In Vivo Studies

#### 3.1.1. Effect of KYP-2047 on Tumor Growth

**Figure 1:**
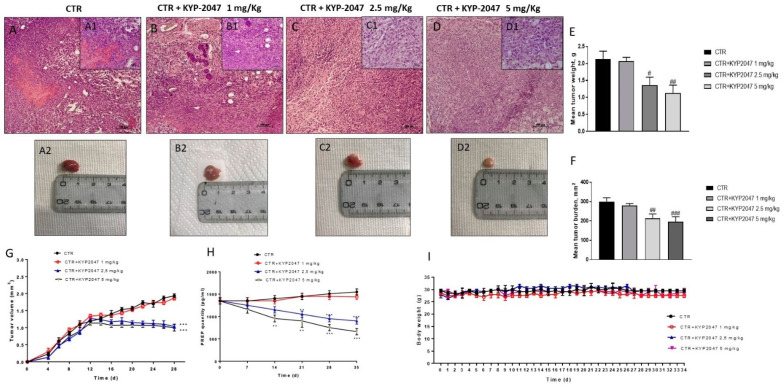
Effect of KYP-2047 on tumor growth. An elevated tumor mass was observed in the control group (**A**) while the treatment with KYP-2047 at doses of 2.5 mg/kg and 5 mg/kg significantly reduced tumor mass and neutrophil infiltration (**C**,**D**) more than KYP-2047 at dose of 1 mg/kg (**B**). Moreover, the panel (**E**,**F**) showed a reduction in tumor volume and tumor weight respectively following KYP-2047 treatment at doses of 2.5 mg/kg and 5 mg/kg without encountering important weight differences (Panel **I**). Additionally, the panel H showed a decrease of PREP expression following KYP-2047 treatment particularly from day 14. Data are representative of at least three independent experiments. Sections were observed and photographed at 10x magnification. (**E**) # *p* < 0.05 vs. CTR; ## *p* < 0.01 vs. CTR; (**F**) ## *p* < 0.01 vs. CTR; ### *p*< 0.001 vs. CTR. (**G**) *** *p* < 0.001 vs. CTR. (**H**) ** *p* < 0.01 vs. CTR; *** *p* < 0.001 vs. CTR.

The histological analysis of the control group (Figure 1A) showed a significant subcutaneous tumor mass, associated to an increase in necrosis and neutrophil infiltration; while the treatment with KYP-2047 at doses of 2.5 mg/kg and 5 mg/kg showed a reduction in tumor sections as well as neutrophil infiltration (Figure 1C,D), much more than KYP-2047 at the dose of 1 mg/kg (Figure 1B). Furthermore, we observed a marked reduction of mean tumor burden, tumor volume and tumor weight following KYP-2047 treatment at doses of 2.5 mg/kg and 5 mg/kg, much more than KYP-2047 1 mg/kg (Figure 1E–G). Moreover, to better understand if the expression levels of PREP changed during the course of treatment in the tumors, we decided to verify the expression of PREP during the treatment with KYP-2047 by ELISA kit. The results showed that KYP-2047 at doses of 2.5 mg/kg and 5 mg/kg was able to reduce significantly PREP levels particularly from day 14 (Figure 1H). During the course of treatment, no important change in animals’ weight was seen (Figure 1I).

#### 3.1.2. Effect of KYP-2047 on Angiogenesis 

**Figure 2:**
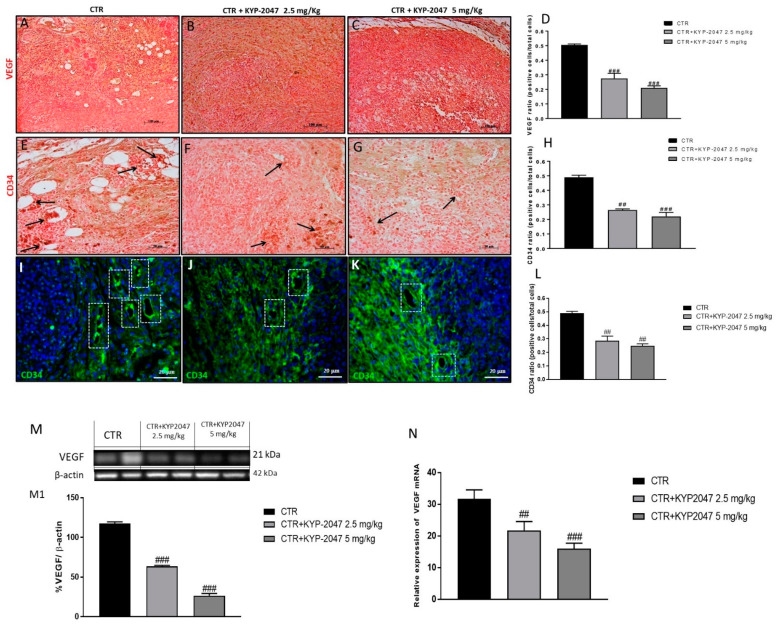
Effect of KYP-2047 on vascular endothelial-growth-factor (VEGF) and CD34 expression. Immunohistochemical staining showed a marked expression of VEGF and CD34 in the control group (**A**,**E**) whereas the treatment with KYP-2047 at doses of 2.5 mg/kg and 5 mg/kg significantly reduced their expression (**B**,**C**,**F**,**G**). Sections were observed and photographed at 10×, 20× and 40× magnification The data for VEGF were confirmed also by western blot analysis and quantitative real-time polymerase chain reaction (RT-qPCR), showing a decrease of VEGF expression following KYP-2047 treatment (**M**,**N**). Moreover, the data for CD34 were confirmed also by immunofluorescence assay (**I**,**J**,**K**). Data are representative of at least three independent experiments. (**D**) ### *p* < 0.001 vs. CTR; (**H**) ## *p* < 0.01 vs. CTR; ### *p* < 0.001 vs. CTR. (**L**) ## *p* < 0.01 vs. CTR; (**M**) ### *p* < 0.001 vs. CTR. (**N**) ## *p* < 0.01 vs. CTR; ### *p* < 0.001 vs. CTR.

**Figure 3:**
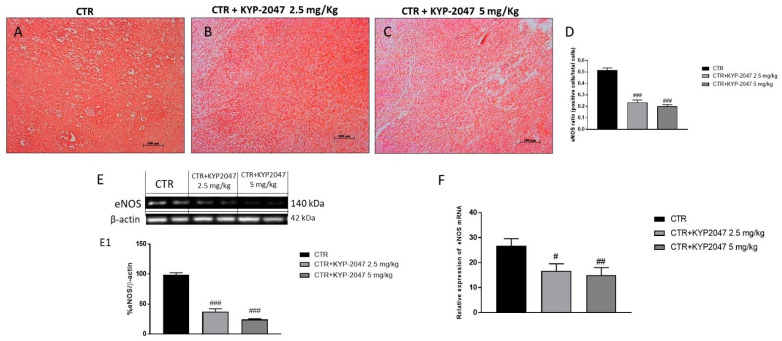
Effect of KYP-2047 on endothelial-nitric-oxide synthase (eNOS) expression. Immunohistochemical staining showed a marked expression of eNOS in the control group (**A**) whereas the treatment with KYP-2047 at doses of 2.5 mg/kg and 5 mg/kg significantly reduced its expression (**B**,**C**). Sections were observed and photographed at 10× magnification. The data were confirmed by Western blot analysis and RT-qPCR, showing a decrease of eNOS expression following KYP-2047 treatment (**E**,**F**). Data are representative of at least three independent experiments. (**D**) ### *p* < 0.001 vs. CTR; (**E**) ### *p* < 0.001 vs. CTR. (**F**) # *p* < 0.05 vs CTR; ## *p* < 0.01 vs. CTR.

Angiogenesis is an essential process for tumor growth [22]. GB is characterized by a deregulation of angiogenic growth factors as VEGF and eNOS expression, which play a key role in maintaining vascular homeostasis and vessel integrity [22,23,24]. Therefore, in this study we decided to investigate by immunohistochemical staining the levels of VEGF and eNOS. Our results demonstrated a significant increase of VEGF and eNOS levels in the control group (Figure 2A and Figure 3A respectively); however, the treatment with KYP-2047 at doses of 2.5 mg/kg and 5 mg/kg significantly reduced their expression (Figure 2B,C, see immunohistochemistry score Figure 2D; Figure 3B,C, see immunohistochemistry score Figure 3D respectively) in a dose-dependent manner. These results were confirmed also by Western blot analysis and RT-qPCR, showing a significantly reduction of VEGF and eNOS expression in the groups treated with KYP-2047 at doses of 2.5 mg/kg and 5 mg/kg compared to control group (Figure 2M, see densitometric analysis Figure 2M1,N and Figure 3E, see densitometric analysis Figure 3E1,F). 

Additionally, we evaluated the expression of CD34, a transmembrane glycoprotein involved in the process of newly-forming tumour vessels [25] by immunohistochemistry and immunofluorescence analysis. In this context, our results showed a significant reduction of CD34 expression in the groups treated with KYP-2047 at doses of 2.5 mg/kg and 5 mg/kg compared to control group (Figure 2E–G; see immunohistochemistry score Figure 2H) (Figure 2I–K; see CD34 ratio positive cells score Figure 2L). 

**Figure 4:**
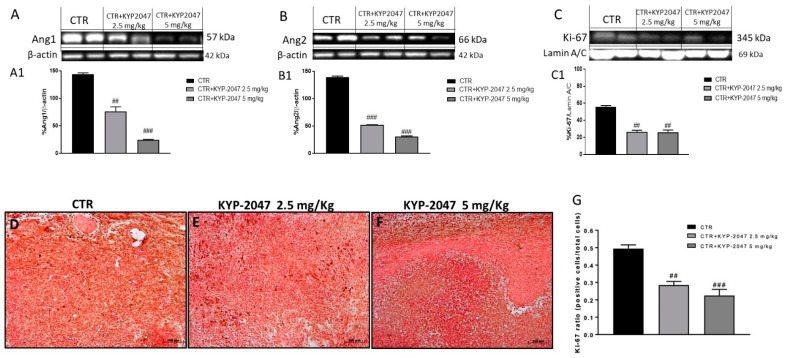
Effect of KYP-2047 on Ang1, Ang2 and Ki-67 expression. The blots revealed a significant increase of Ang1 and Ang2 expression in the control group while the treatment with KYP-2047 at doses of 2.5 mg/kg and 5 mg/kg significantly reduced their expression (**A**,**B**). Sections were observed and photographed at 10× magnification Moreover, the panel (**C**) revealed a significant increase of Ki-67 in the control group while the treatment with KYP-2047 at doses of 2.5 mg/kg and 5 mg/kg significantly decreased its expression. The data for Ki-67 was confirmed also by immunohistochemistry (**D**–**F**). Data are representative of at least three independent experiments. (**A**) ## *p* < 0.01 vs. CTR; ### *p* < 0.001 vs. CTR; (**B**) ### *p* < 0.01 vs. CTR; (**C**) ## *p* < 0.01 vs. CTR. (**G**) ## *p* < 0.01 vs. CTR; ### *p* < 0.001 vs. CTR.

Studies on angiogenesis have emphasized the importance of others angiogenic factors involved in tumor growth such as angiopoietins, in particular angiopoietin 1 (Ang1) and angiopoietin 2 (Ang2), currently proposed as biomarkers of GB [26,27]. Therefore, we detected Ang1 and Ang2 expression by Western blot analysis on tumor samples. Our results showed a significantly decrease of Ang1 and Ang2 levels following KYP-2047 treatment at doses of 2.5 mg/kg and 5 mg/kg compared to control group (Figure 4A, see densitometric analysis 4A1; Figure 4B, see densitometric analysis 4B1) in a dose-dependent manner. 

Furthermore, we investigated the role of Ki-67, a nuclear protein associated with tumor proliferation and progression [28,29]. As shown in the Figure 4C, the blot revealed a marked expression of Ki-67 in the control group whereas the treatment with KYP-2047 at doses of 2.5 mg/kg and 5 mg/kg significantly reduced its expression (see densitometric analysis 4C1). Moreover, Ki-67 was evaluated also by immunohistochemistry assay confirming the results obtained as showed in the Figure 4D–F (see immunohistochemistry score 4G). 

#### 3.1.3. Effect of KYP-2047 on Apoptosis Pathway 

**Figure 5:**
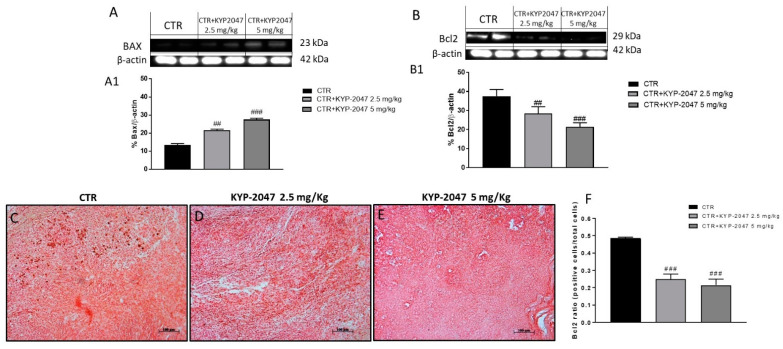
Effect of KYP-2047 on apoptosis pathway in the U87-xenograft model. The blots revealed an increase of pro-apoptotic Bax expression and a decrease of Bcl2 expression following KYP-2047 treatment compared to control group (**A**,**B**). Additionally, immunohistochemistry staining confirmed a decrease of Bcl2 expression after KYP-2047 treatment. (**C**–**F**). Sections were observed and photographed at 10× magnification. Data are representative of at least three independent experiments. (**A**) ## *p* < 0.01 vs. CTR; ### *p* < 0.001 vs. CTR; (**B**) ## *p* < 0.01 vs. CTR; ### *p* < 0.001 vs. CTR; (**F**) ### *p* < 0.001 vs. CTR.

**Figure 6:**
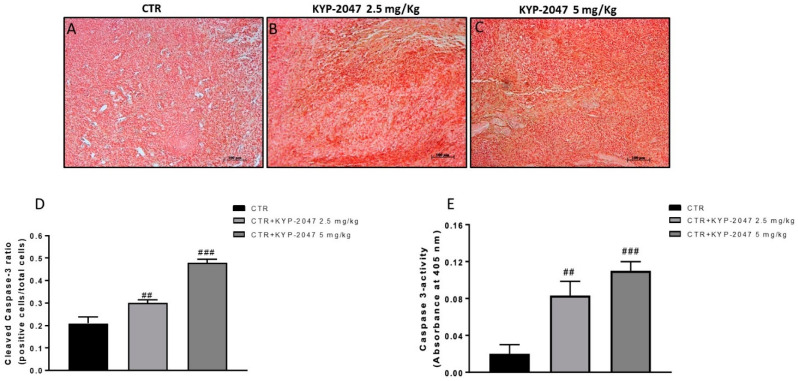
Effect of KYP-2047 on caspase-3 expression. Immunohistochemistry assay revealed an increase of cleaved-caspase-3 expression following KYP-2047 treatment at doses of 2.5 mg/kg and 5 mg/kg compared to control group (**A**–**C**). Sections were observed and photographed at 10× magnification. Additionally, the data for caspase-3 were confirmed also by a colorimetric assay kit as shown in the panel E. Data are representative of at least three independent experiments. (**D**) ## *p* < 0.01 vs. CTR; ### *p* < 0.001 vs. CTR; (**E**) ## *p* <0.01 vs. CTR; ### *p* < 0.001 vs. CTR.

Considering the key role of apoptosis in GB progression [30], we evaluated the pro-apoptotic Bax, and anti-apoptotic Bcl2 protein by western blot analysis on tumor samples. The results showed that KYP-2047 was able to increase Bax expression and reduce Bcl2 expression (Figure 5A; see densitometric analysis 5A1; Figure 5B, see densitometric analysis 5B1). Moreover, the ability of KYP-2047 to modulate Bcl2 expression was confirmed by immunohistochemistry as shown in Figure 5C–E (see immunohistochemistry score Figure 5F). Furthermore, we detected caspase-3 levels by immunohistochemistry and by a colorimetric assay kit on tumor samples, showing that KYP2047 at doses of 2.5 and 5 mg/kg significantly increased caspase-3 activity compared to the control group (Figure 6A–C; see immunohistochemistry score Figure 6D,E

### 3.2. In Vitro Studies

#### 3.2.1. Effect of KYP-2047 on Cell Viability

**Figure 7:**
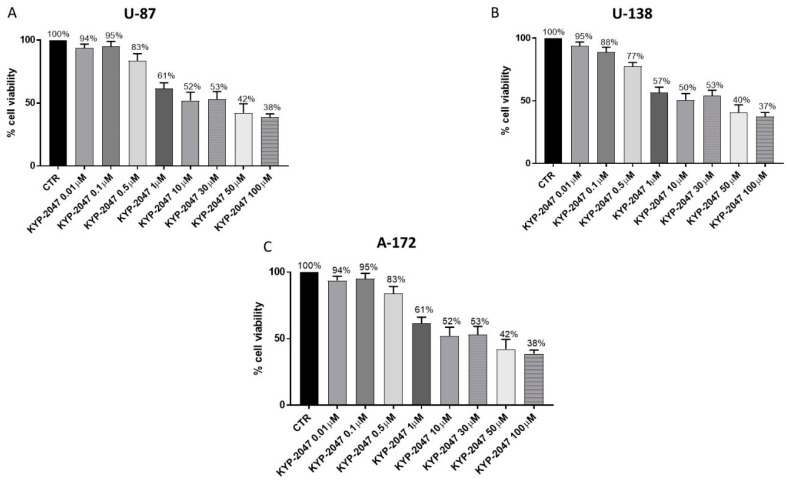
Effect of KYP-2047 on U-87, U-138 and A-172 cell viability. Cell viability was evaluated using MTT assay 24 h after KYP-2047 treatment at the concentrations of 0.01 μM, 0.1 μM, 0.5 μM, 1 μM, 10 μM, 30 μM, 50 μM and 100 μM. U-87, U-138 and A-172 cells showed a similar decrease of cell viability following KYP-2047 treatment in a concentration-dependent manner (**A**–**C**). Data are representative of at least three independent experiments.

KYP-2047 cytotoxicity was evaluated incubating U-87, A-172 and U-138 cells with growing concentrations of KYP-2047 (0.01 μM, 0.1 μM, 0.5 μM, 1 μM, 10 μM, 30 μM, 50 μM and 100 μM) for 24 h. KYP-2047 treatment showed a significant decrease of cell viability in all three cell lines in a concentration dependent-manner as shown in the Figure 7A–C. Therefore, based on MTT results, we decided to continue testing for other analysis only KYP-2047 at concentrations of 50 μM and 100 μM on U-87, A172 and U138 cells because they represented the most cytotoxic concentrations.

#### 3.2.2. Effect of KYP-2047 on Apoptosis Pathway

**Figure 8:**
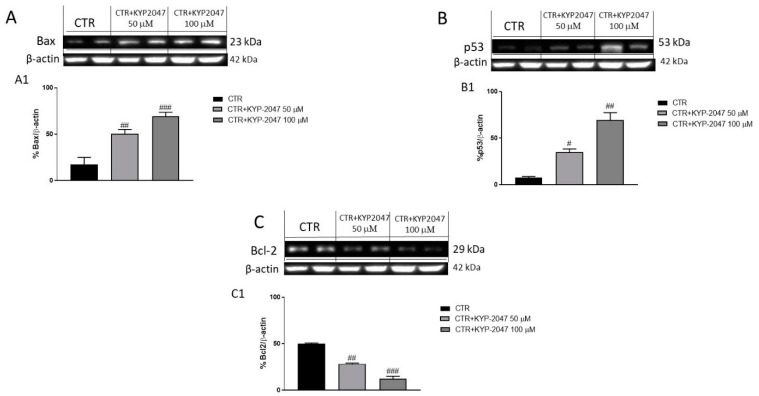
Effect of KYP-2047 on apoptosis pathway in U-87 cell lysates. The blots on U87 cell lysates revealed an increase of pro-apoptotic Bax and p53 expression following KYP-2047 treatment at the concentrations of 50 μM and 100 μM compared to control group (**A**,**B**). Moreover, KYP-2047 at the concentrations of 50 μM and 100 μM reduced significantly Bcl2 expression compared to control group (**C**). Data are representative of at least three independent experiments. (**A**) ## *p* < 0.01 vs. CTR; ### *p* < 0.001 vs. CTR; (**B**) # *p* < 0.05 vs. CTR; ## *p* < 0.01 vs. CTR; (**C**) ## *p* < 0.01 vs. CTR; ### *p* < 0.001 vs. CTR.

Apoptosis plays a key role in the development of cancer including GB [31]. Deregulation of apoptotic process is a relevant hallmark of a tumor [31], responsible not only for its progression but also for tumor resistance to therapies [32]. Therefore, we investigated the effect of KYP-2047 on the apoptosis pathway in U-87, A-172 and U-138 cell lysates evaluating the pro-apoptotic Bax, tumor suppressor p53 and anti-apoptotic Bcl2 protein by Western blot analysis (The original Western blot can be found in Figure S5). Our results revealed an increase of Bax and p53 levels following KYP-2047 treatment in U87 cell lysates performed for 24 h at the concentrations of 50 μM and 100 μM compared to control group (Figure 8A, see densitometric analysis 8A1; Figure 8B, see densitometric analysis 8B1, respectively); while Bcl2 expression was significantly reduced following KYP-2047 treatment compared to control group (Figure 8C; see densitometric analysis 8C1) in a concentration-dependent manner. The same results appear for A-172 and U-138 cell lysates, confirming an increase of pro-apoptotic Bax and p53 expression following KYP-2047 treatment compared to control group (Figure S1A, see densitometric analysis 1A1; Figure S1B, see densitometric analysis 1B1, respectively) (Figure S2A, see densitometric analysis 2A1; Figure S2B, see densitometric analysis 2B1, respectively) and a decrease of anti-apoptotic Bcl2 protein expression (Figure S1C; see densitometric analysis 1C) (Figure S2C; see densitometric analysis 2C1). 

#### 3.2.3. Effect of KYP-2047 on TGF-β and Caspase-3 Expression by Immunofluorescence Assay 

**Figure 9:**
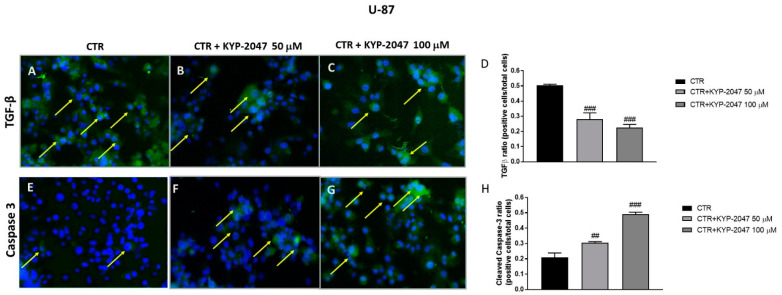
Effect of KYP-2047 on transforming growth factor-β (TGF-β) and caspase-3 expression in U-87 cells. Immunofluorescence assay performed on U-87 cells revealed a marked expression of TGF-β in the control group (**A**), while the treatment with KYP-2047 at the concentrations of 50 μM and 100 μM reduced significantly TGF-β expression (**B**,**C**). Additionally, immunofluorescence staining showed an increase of caspase-3 levels in the groups treated with KYP-2047 at the concentrations of 50 μM and 100 μM (**F**,**G**) compared to control group (**E**). Data are representative of at least three independent experiments. (**D**) ### *p* < 0.001 vs. CTR; (**H**) ## *p* < 0.01 vs. CTR; ### *p* < 0.001 vs. CTR.

Current studies have focused on the role of TGF-β in the tumor microenvironment suggesting that it plays a key role for GB progression [33,34]. Therefore, we investigated TGF-β expression by immunofluorescence assay on U-87, A-172 and U-138 cell lines. Our results confirmed a significant reduction of TGF-β expression after KYP-2047 treatment at the concentrations of 50 μM and 100 μM compared to the control group in U-87 cells (Figure 9A–C, see TGF-β ratio positive cells score Figure 9D), as well as in A-172 and U-138 cell lines (Figure S3A–C; see TGF-β ratio positive cells score 3D); (Figure S4A–C; see TGF-β ratio positive cells score 4D). 

In addition to the regulation of the cell cycle and differentiation, TGF-β is able to induce apoptosis [35] promoting the activation of pro-apoptotic caspase-3, a member of the cysteine-aspartic acid protease family [36]. Thus, in this study we detected caspase-3 expression by immunofluorescence assay in all three GB cell lines. The results obtained showed an increase of caspase-3 expression following KYP-2047 treatment at the concentrations of 50 μM and 100 μM compared to the control group in U-87 cells (Figure 9E–G, see caspase-3 ratio positive cells score 9H) as well as in A-172 and U-138 cell lines in a concentration-dependent manner (Figure S3E–G; see caspase-3 ratio positive cells score 3H); (Figure S4E–G; see caspase-3 ratio positive cells score 4H).

## 4. Discussion 

Glioblastoma (GB) is the most common and aggressive primary brain tumor in adults [37]. GB arise from glial cells but can also develop from astrocytic or neural stem/progenitor cells [38]. GB can be classified into primary and secondary subtypes, based on pre-existing lesion [38]. The primary GB subtype develops rapidly de novo in elderly patients without clinical or histologic evidence, whereas the secondary subtype develops from evolution of low-grade astrocytic tumours over the course of 4–5 years [38]. In the last decade, many studies have focused on the role of genetic mutations which contribute to GB initiation as TP53 and isocitrate dehydrogenase (IDH) mutations [37,39]. GB is characterized by a high degree of invasiveness, cell proliferation, angiogenesis and apoptosis alteration [2]. Despite scientific advances, the survival rate for patients with GB remains low and additional therapies are needed [12]. Previous studies have demonstrated that angiogenesis and apoptosis play a key role in GB pathology promoting cell survival and proliferation [10,31]. Therefore, the modulation of angiogenesis and apoptosis processes represent a valid strategy to counteract or reduce GB progression. KYP-2047 (4-phenylbutanoyl-L-prolyl-2(S)-cyanopyrrolidine) was developed as a highly specific and potent POP (or PREP) inhibitor, a serine protease involved in the angiogenesis process [40]. Recent studies revealed that KYP-2047 was able to modulate not only angiogenesis [11] but also cell cycle and differentiation [12,13]. Therefore, in this study we investigated the potential effect of KYP-2047 on angiogenesis and apoptosis pathways in an in vivo U87-xenograft model and in vitro study on the human GB cell line. 

Firstly, we evaluated the ability of KYP-2047 to inhibit tumor growth in the xenograft model. Our results showed a high-grade necrosis and neutrophil infiltration in the control group, while KYP-2047 at higher doses significantly reduced subcutaneous tumor mass as well as neutrophil infiltration. Moreover, KYP-2047 significantly decreased mean tumor burden and tumor weight at higher doses, without encountering important weight differences. 

Interestingly, treatment with KYP-2047 was able to reduce. PREP levels in serum of animals, particularly from day 14.

GB is one of the most highly angiogenic solid tumor [41]. Its tumor vasculature is both structurally and functionally abnormal, characterized by a dense network of vessels tortuous with increased diameter and thickened basement membranes [41]. Thus, angiogenesis is considered as a pathologic hallmark of GB, leading to VEGF activation, an angiogenic growth factor that promotes glioblastoma proliferation and CD34 activation, a transmembrane glycoprotein involved in the process of newly-forming tumour vessels [25,42]. Therefore, in this study we investigated VEGF and CD34 expression, showing that KYP-2047 at higher doses was able to reduce their expression significantly compared to the control group. Moreover, we investigated the role of eNOS, a relevant endothelial enzyme that modulates vascular homeostasis and vessel integrity [24]. In this context, our results showed that the control group was characterized by an increase of eNOS expression, whereas KYP-2047 significantly reduced eNOS expression. 

The formation of new blood vessels is an essential process for GB growth [22]. In addition to VEGF, this process requires the involvement of other angiogenic factors as the angiopoietins, in particular angiopoietin 1 (Ang1) and angiopoietin 2 (Ang2) which have similar functions [26,43]. Previous studies revealed that Ang1 and Ang2 regulate vascular development and remodelling, promoting tumor growth [43,44]. Therefore, we decided to investigate the expression of Ang1 and Ang2 in GB; our results showed that the control group was characterized by an increase of Ang1 and Ang2 expression, while the treatment with KYP-2047 was able to significantly reduce their expression, inhibiting GB proliferation. 

An increased vascularization provides to the tumor cells more oxygen and nutrients, promoting metastatic spread and cell proliferation [22]. In this context, Mastronardi et al. evaluated the correlation between angiogenesis and proliferation processes, through Ki-67 evaluation, a nuclear protein that regulates the cell cycle and differentiation [45]. Ki-67 is considered a relevant marker of tumor proliferation in GB [45,46]. Thus, we decided to evaluate Ki-67 expression, demonstrating that the control group was characterized by an increase of Ki-67 level, while KYP-2047 treatment was able to significantly reduce its expression. 

Moreover, considering the key role of apoptosis in GB progression [3], we decided to investigate Bax, Bcl2 and caspase-3 expression in the U87-xenograft model, showing that KYP-2047 at doses of 2.5 mg/kg and 5 mg/kg was able to increase pro-apoptotic Bax and caspase-3 expression while Bcl2 expression was significantly reduced following KYP-2047 treatment. 

To confirm the promising results obtained by an in vivo U87-xenograft model, we decided to conduct an in vitro model of GB on U-87, A-172 and U-138 cell lines. Firstly, we evaluated the cytotoxicity of KYP-2047 at different concentrations on U-87, A-172 and U-138 GB cells, demonstrating that KYP-2047 was able to significantly reduce cell viability in all three GB cell lines in a concentration-dependent manner. 

Previous studies revealed that also apoptosis plays a key role in the development of GB [31,47]. It has been demonstrated that a down-regulation of apoptosis is associated with tumor survival [31,47]. Therefore, in this study we decided to evaluate the effect of KYP-2047 on the apoptosis pathway by evaluating protein levels of pro-apoptotic Bax, p53 and anti-apoptotic Bcl2 on U-87, A-172 and U-138 cell lysates. Our results revealed that KYP-2047 reduced Bcl2 expression, while Bax and p53 expression were significantly increased following KYP-2047 treatment in a concentration-dependent manner in all three GB cell lines, confirming apoptosis modulation. 

Tumor proliferation is associated with an increase of TGF-β expression [48]. 

TGF-β regulates cell differentiation and apoptosis, promoting caspase-3 activation, a key regulator in apoptotic pathway [36]. Thus, we investigated the expression of TGF-β and caspase-3 in the in vitro model [49].

The results showed an increase of TGF-β expression in the control group while KYP-2047 treatment significantly reduced its expression in U-87, A-172 and U-138 cell lines. In addition, a marked increase of pro-apoptotic caspase-3 expression was revealed following KYP-2047 treatment, highlighting the ability of KYP-2047 to modulate apoptosis in all three GB cell lines. 

Thus, the results obtained in an in vivo xenograft model and in an in vitro study on human GB cell lines revealed that KYP-2047 was able to reduce GB progression and growth by modulating angiogenesis and apoptosis pathways. Therefore, KYP-2047 could be considered as an alternative therapeutic strategy to counteract or reduce GB progression. 

## 5. Conclusions

The data obtained revealed the ability of KYP-2047 to modulate angiogenesis and apoptosis pathways in an in vivo xenograft model and in an in vitro model of GB, reducing tumor progression. Therefore, on the basis of these results, KYP-2047 could represent an available strategy for the treatment of GB. 

## Data Availability

All data generated or analyzed during this study are included in this article.

## Supplementary Materials

The following are available online at https://www.mdpi.com/article/10.3390/cancers13143444/s1, Figure S1: Effect of KYP-2047 on apoptosis pathway in A-172 cell lysates., Figure S2: Effect of KYP-2047 on apoptosis pathway in U-138 cell lysates., Figure S3: Effect of KYP-2047 on TGF-β and Caspase3 expression in A-172 cells., Figure S4: Effect of KYP-2047 on TGF-β and Caspase3 expression in U-138 cells., Figure S5: Original western blot.
