# Supplementary material for: KYP-2047, an Inhibitor of Prolyl-Oligopeptidase, Reduces GlioBlastoma Proliferation through Angiogenesis and Apoptosis Modulation
Source: Cancers (Basel). 2021 Jul 9;13(14):3444. doi: 10.3390/cancers13143444 (PMC8306782; doi:10.3390/cancers13143444)
Supplement: Supplementary file 1 [file cancers-13-03444-s001.zip › cancers-1255044-supplementary.pdf]

cancers

Supplementary Material: KYP-2047, an Inhibitor of Prolyl-Oligopeptidase, Reduces GlioBlastoma Proliferation through Angiogenesis and Apoptosis Modulation

Sarah Adriana Scuderi, Giovanna Casili, Alessio Ardizzone, Stefano Forte, Lorenzo Colarossi, Serena Sava, Irene Paterniti, Emanuela Esposito, Salvatore Cuzzocrea and Michela Campolo

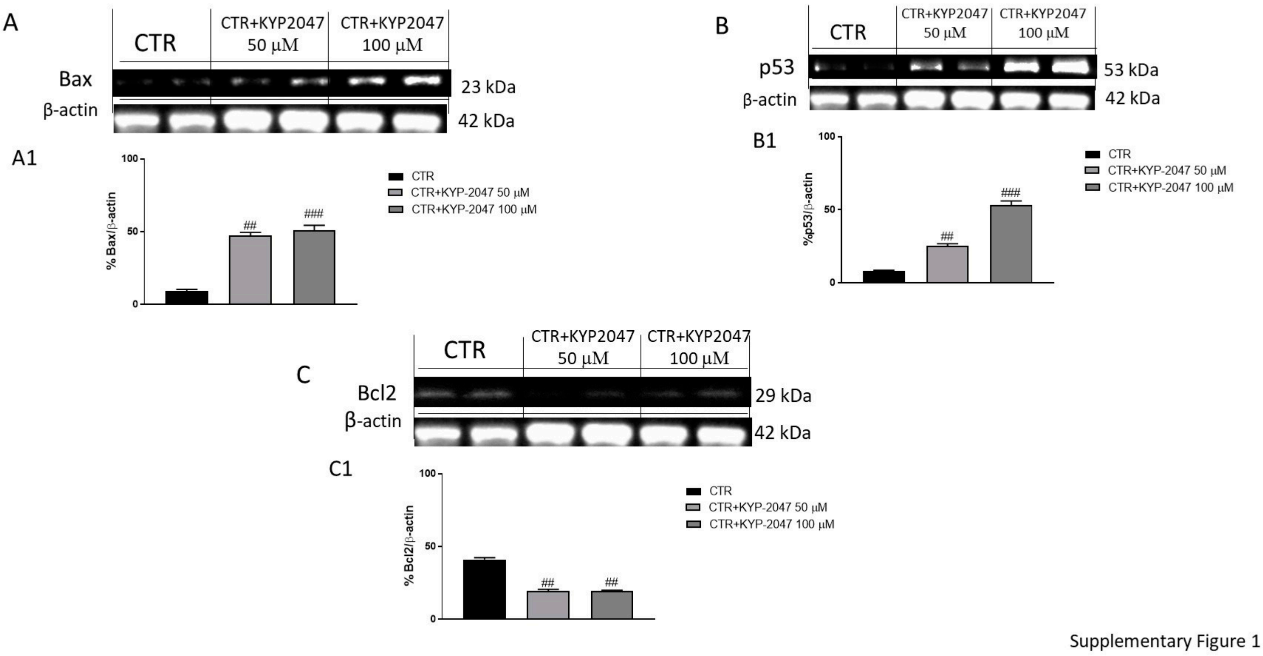

Figure S1. Effect of KYP-2047 on apoptosis pathway in A-172 cell lysates. The blots on A172 cell lysates showed an increase of Bax and p53 expression following KYP-2047 treatment at the concentrations of 50 μM and 100 μM compared to control group (**A**,**B**). Additionally, KYP-2047 at the concentrations of 50 μM and 100 μM reduced significantly Bcl2 expression compared to control group (**C**). Data are representative of at least three independent experiments. (**A**) ## $p < 0.01$ vs CTR; ### $p < 0.001$ vs. CTR; (**B**) ## $p < 0.01$ vs. CTR; ### $p < 0.001$ vs. CTR; (**C**) ## $p < 0.01$ vs. CTR.

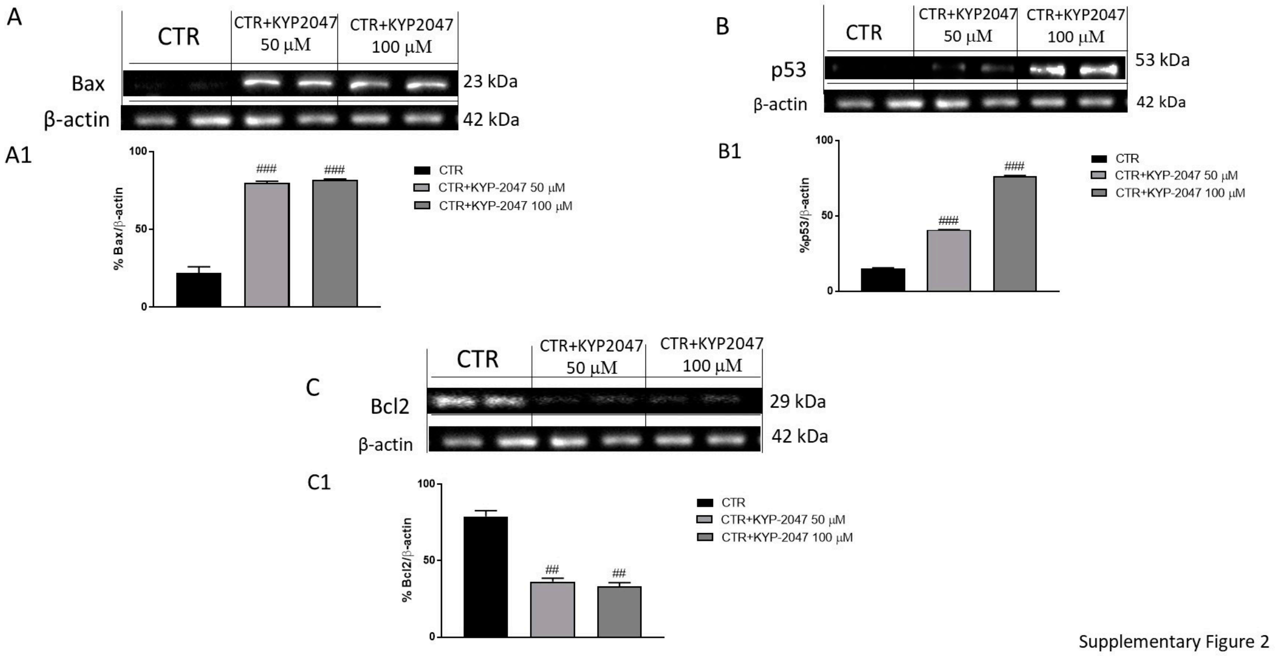

Supplementary Figure 2

Figure S2. Effect of KYP-2047 on apoptosis pathway in U-138 cell lysates. The blots on U-138 cell lysates showed an increase of pro-apoptotic Bax and p53 expression after KYP-2047 treatment at the concentrations of 50 μM and 100 μM compared to control group (**A**,**B**). Whereas, KYP-2047 at the concentrations of 50 μM and 100 μM reduced significantly Bcl2 expression compared to control group (**C**). Data are representative of at least three independent experiments. (**A**) ### $p < 0.001$ vs CTR; (**B**) ### $p < 0.001$ vs. CTR; (**C**) ## $p < 0.01$ vs. CTR.

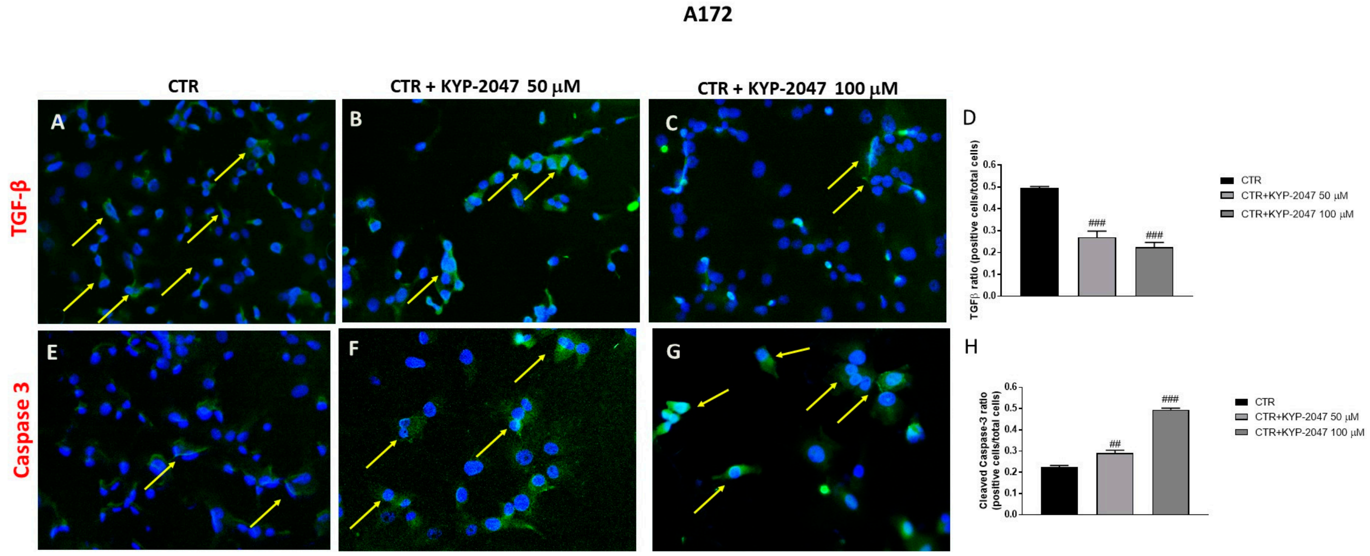

Supplementary Figure 3

Figure S3. Effect of KYP-2047 on TGF-β and Caspase3 expression in A-172 cells. Immunofluorescence assay on A-172 cells revealed an increased expression of TGF-β in the control group (**A**), while the treatment with KYP-2047 at the concentrations of 50 μM and 100 μM reduced significantly TGF-β expression (**B**,**C**). Moreover, immunofluorescence staining showed an increase of Caspase-3 levels in the groups treated with KYP-2047 at the concentrations of 50 μM and 100 μM (**F**,**G**) compared to control group (**E**). Data are representative of at least three independent experiments. (**D**) ### $p < 0.001$ vs. CTR; (H) ## $p < 0.01$ vs. CTR; ### $p < 0.001$ vs. CTR.

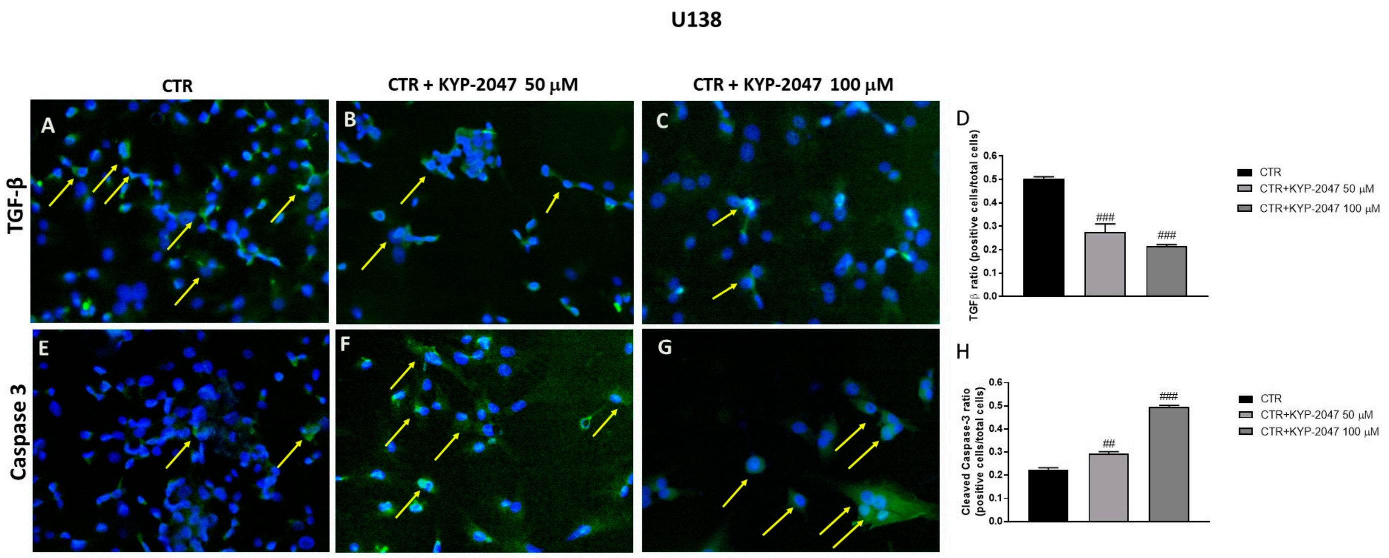

Supplementary Figure 4

Figure S4. Effect of KYP-2047 on TGF-β and Caspase3 expression in U-138 cells. Immunofluorescence assay on U-138 cells showed a marked expression of TGF-β in the control group (**A**), while the treatment with KYP-2047 at the concentrations of 50 μM and 100 μM reduced significantly TGF-β expression (**B**,**C**). Immunofluorescence assay showed an increase of Caspase-3 expression in the groups treated with KYP-2047 at the concentrations of 50 μM and 100 μM (**F**,**G**) compared to control group (**E**). Data are representative of at least three independent experiments. (**D**) ### $p < 0.001$ vs. CTR; (**H**) ## $p < 0.01$ vs. CTR; ### $p < 0.001$ vs. CTR.

Original Western Blot Tumor samples U87-xenograft model

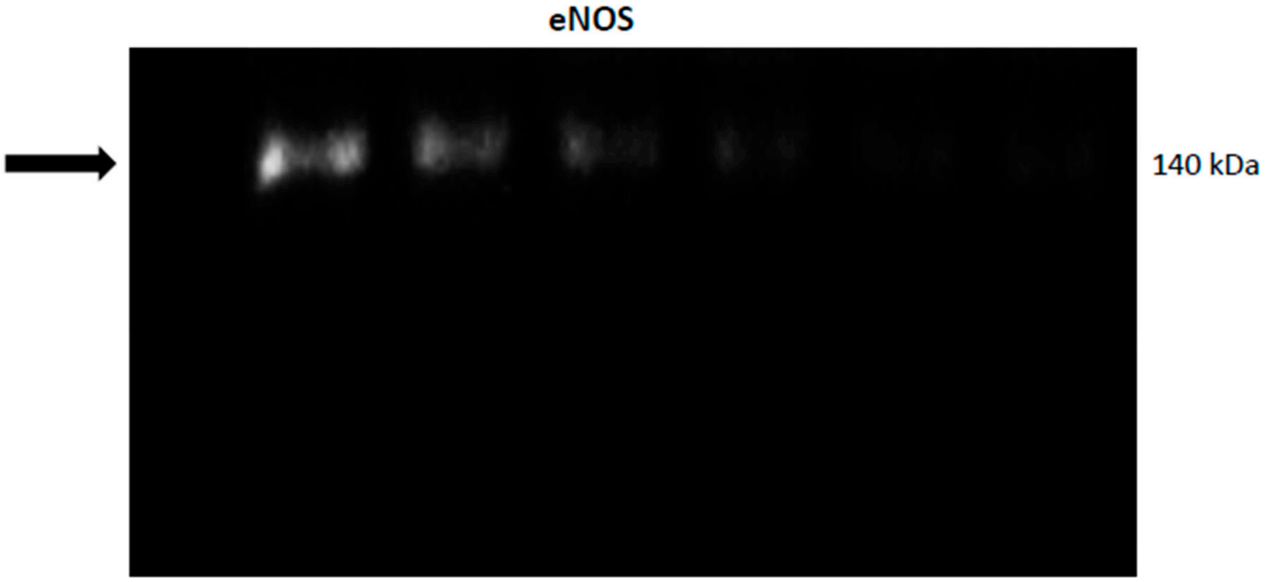

Original Western Blot
Tumor samples U87-xenograft model

VEGF

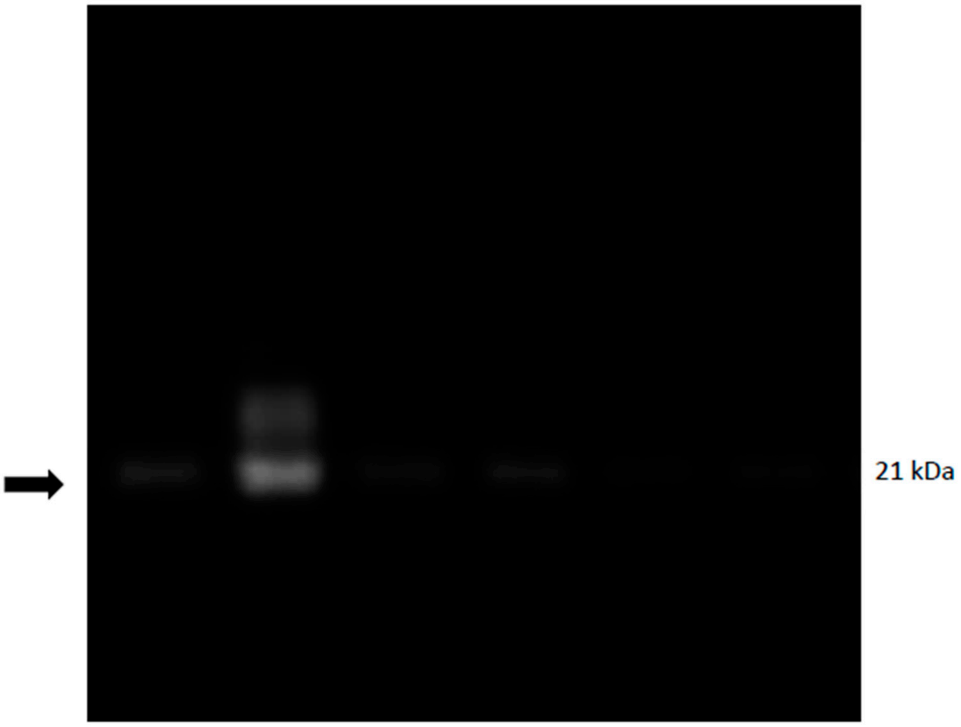

Original Western Blot
Tumor samples U87-xenograft model

Ang1

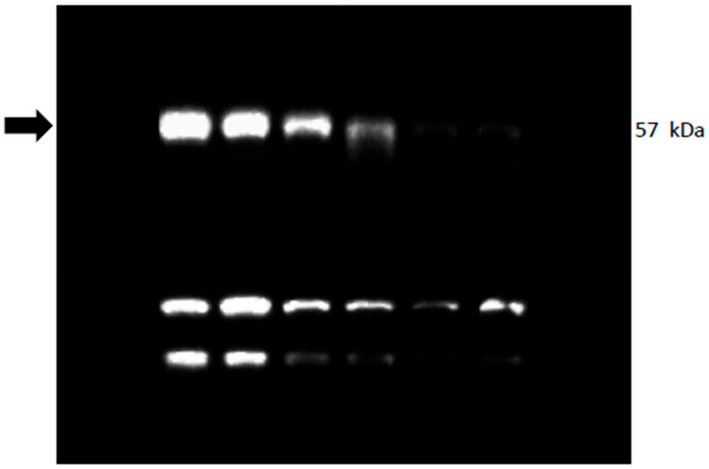

Ang2

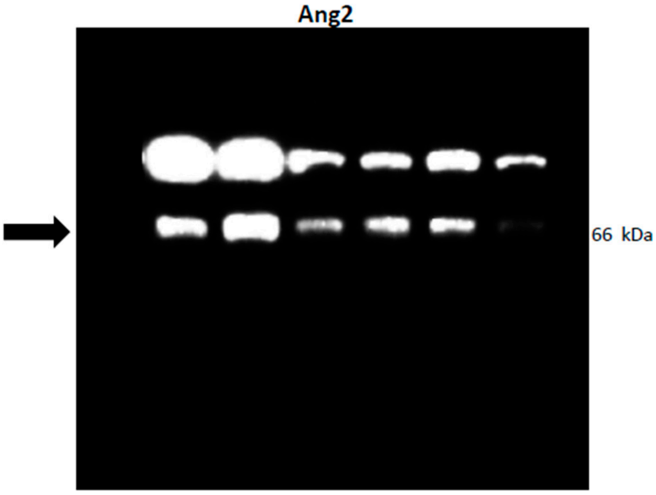

Original Western Blot
Tumor samples U87-xenograft model

β-actin for VEGF, eNOS, Ang1 and Ang2

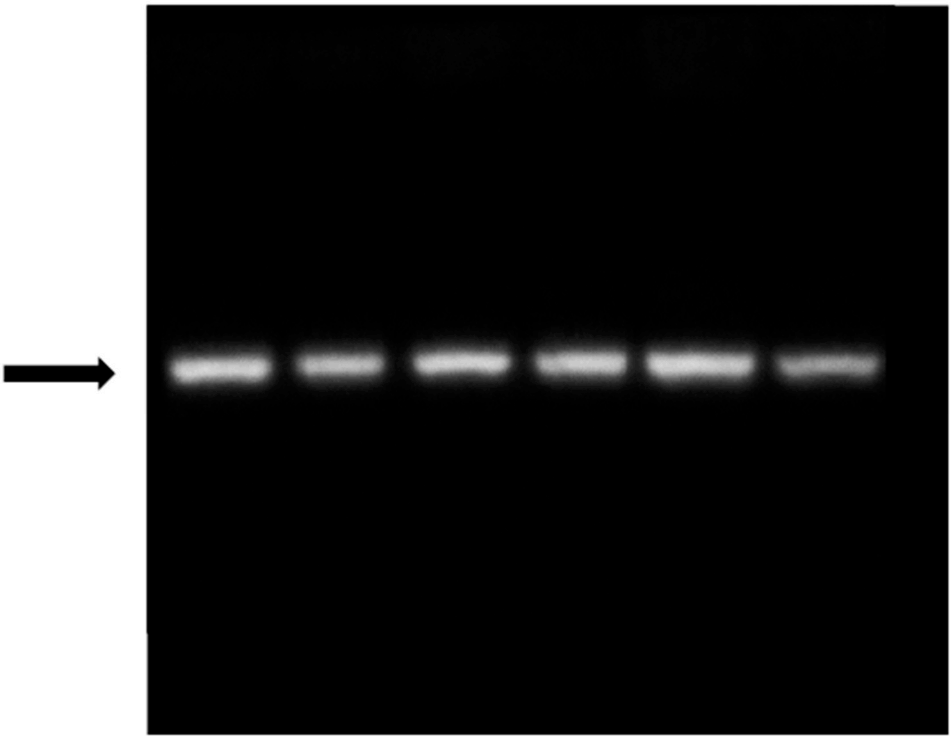

42 kDa

Original Western Blot
Tumor samples U87-xenograft model

Ki-67

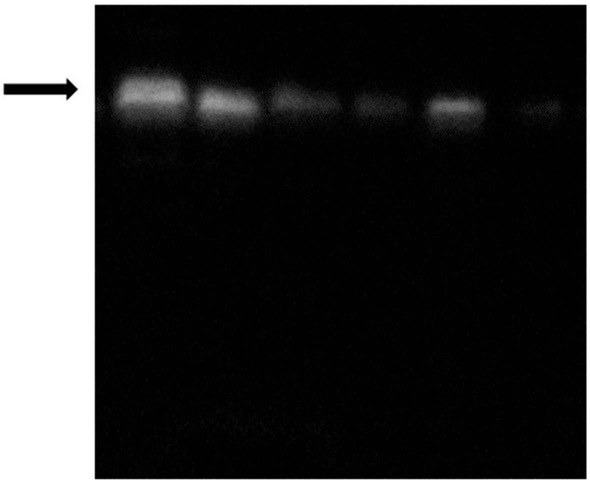

345 kDa

Lamin A/C for Ki-67

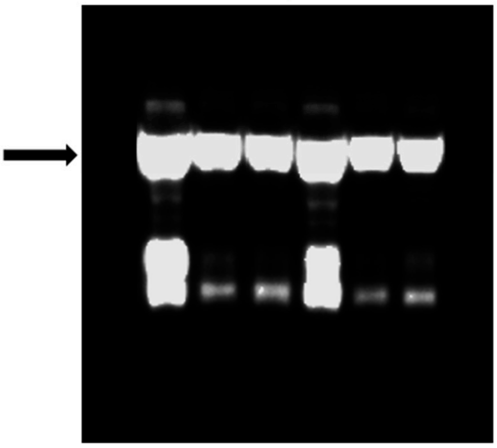

69 kDa

Original Western Blot
Tumor samples U87-xenograft model

Bcl2

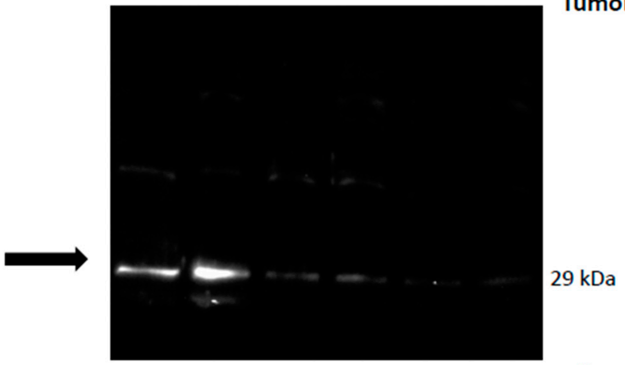

BAX

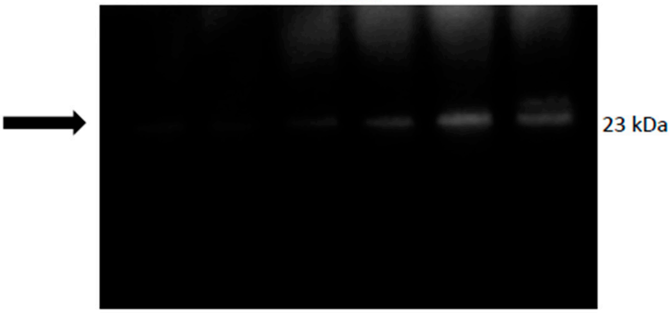

β-actin for Bax and Bcl2

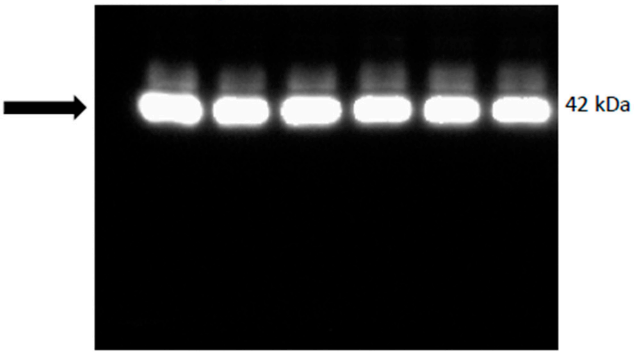

Original Western Blot
U-87 cell lysates

p53

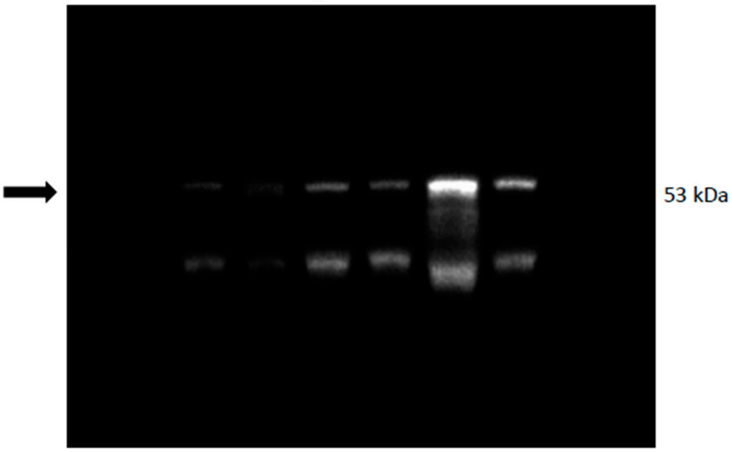

Bax

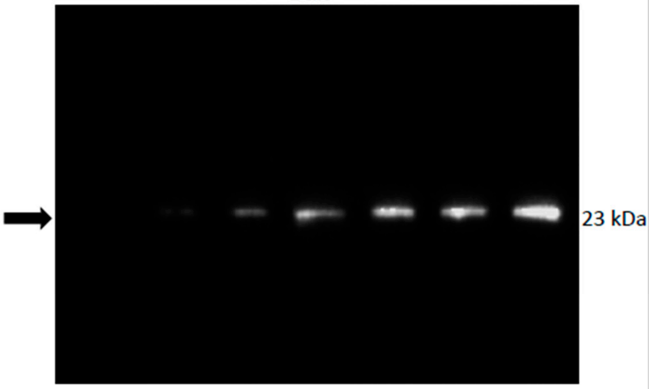

Original Western Blot
U-87 cell lysates

Bcl2

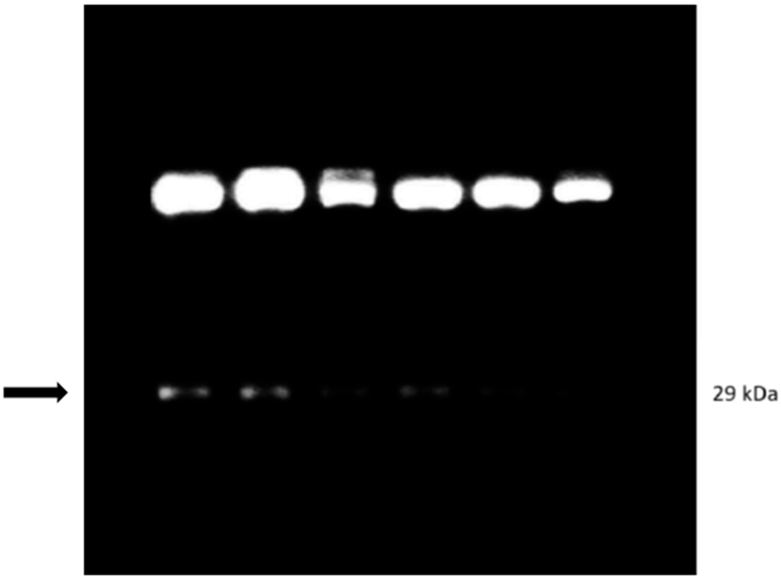

β-actin for Bax, Bcl2 and p53

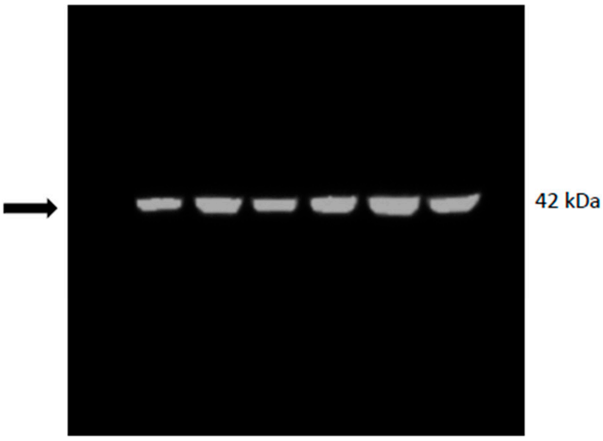

Original Western Blot
A-172 cell lysates

p53

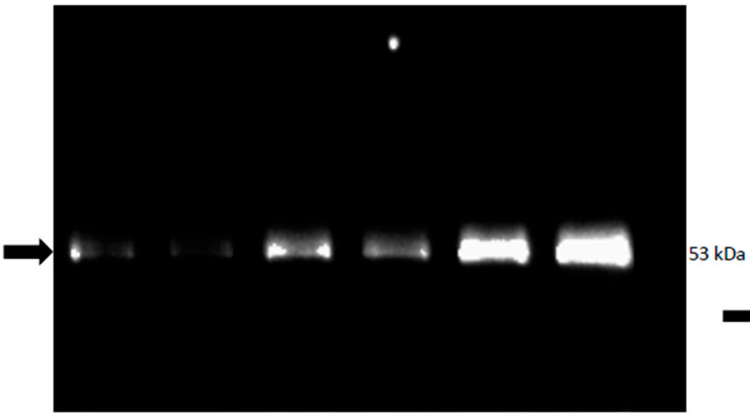

Bax

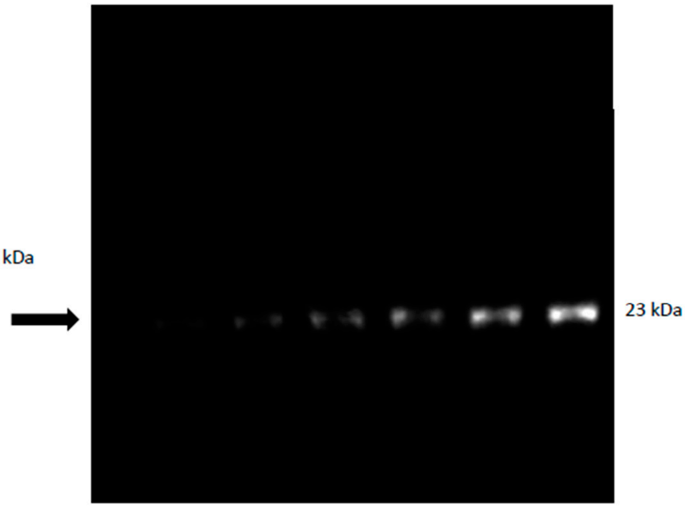

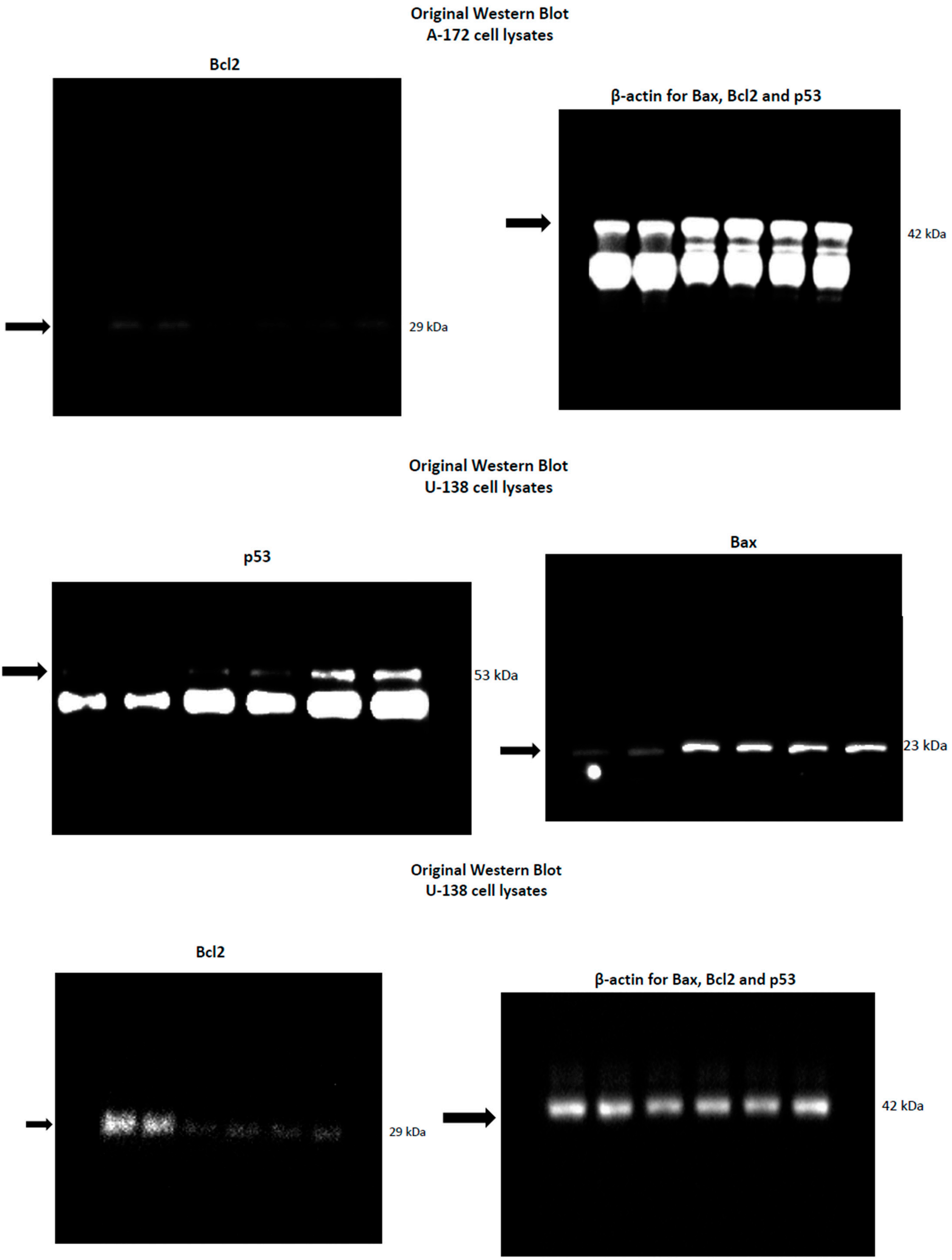

Figure S5. Original Western Blot.
